# 

**Published:** 1940

**Authors:** 


Vol. LVII. No. 216. SUMMER, 1940.

### The Bristol Medico-Chirurgical Journal

PUBLISHED UNDER THE AUSPICES OF
THE BRISTOL MEDICO-CHIRURGICAL SOCIETY.

*Editor:* J. A. NIXON, C.M.G., M.D., F.R.C.P.
WITH WHOM ARE ASSOCIATED
E. W. HEY GROVES, D.Sc., M.S., F.R.C.S.
A. E. ILES, O.B.E., M.B., B.S., F.R.C.S.
A. RENDLE SHORT, M.D., B.S., B.Sc., F.R.C.S.
W. MELVILLE CAPPER, M.B., B.S., F.R.C.S.
T. M. LING, M.D., M.R.C.P.
E. WATSON-WILLIAMS, M.C., Ch.M., F.R.C.S., *Assistant Editor.*

BRISTOL:

J. W. ARROWSMITH LTD., QUAY STREET & SMALL STREET

**Price Three Shillings net.**

PRINTED IN GREAT BRITAIN.

